# The Relationship between Perceived Friendship Quality and Self-Judgements in Adolescent Girls from London

**DOI:** 10.1177/02724316241271327

**Published:** 2024-08-05

**Authors:** Blanca Piera Pi-Sunyer, Jessica Evans, Katy Ratcliffe, Kaushalya Janaarthanan, Saz Ahmed, Willem Kuyken, Tim Dalgleish, Sarah-Jayne Blakemore

**Affiliations:** 1 2152 University of Cambridge, UK; 2 4919 University College London, UK; 3 6396 University of Oxford, UK; 4 47962 MRC Cognition and Brain Science Unit, UK

**Keywords:** adolescence, friendship quality, self-judgements, self-concept, self-appraisal task

## Abstract

Understanding ourselves within our peer environment is an important component of self-development during adolescence, the period of life between the onset of puberty and adulthood (between ages 10 and 24 years). We used a self-appraisal paradigm to investigate cross-sectionally the relationship between perceived friendship quality and self-judgements in adolescent girls. One hundred and sixty-three girls (9–15 years), recruited from London, United Kingdom, rated how well a set of positive and negative adjectives described themselves, or a chosen familiar other. Participants also completed a self-report friendship quality questionnaire. Higher perceived friendship quality predicted lower negative self-judgements and higher positive self-judgements. These relationships did not change across the age range tested, but there was an overall decrease in positivity effect (higher positive judgements compared to negative judgements) with age. These findings highlight the importance of investigating how different components of peer relationships are related to self-concept development in adolescence.

## Introduction

Adolescence is a period of life when belonging to peer groups and social evaluative concerns become particularly salient (Pfeifer & Berkman, 2018; Sawyer et al., 2018; Tomova et al., 2021). During this period, young people are developing their sense of self and use their increasing ability to reflect on the mental states and behaviours of other people (Dumontheil et al., 2010) to learn about their own goals, behaviour and social roles (Crone et al., 2022; Moses-Payne et al., 2021; Sebastian et al., 2008). The quality of peer relationships is a significant determinant of self-worth (Bagwell & Schmidt, 2011; Maunder & Monks, 2019) and adaptive psychosocial functioning in adolescence (van Harmelen et al., 2017, 2021), while peer rejection is associated with increased depression and anxiety symptoms (Pickering et al., 2020). Most socioemotional disorders emerge during adolescence (Blakemore, 2019), and adolescent girls are particularly vulnerable to developing mental health problems (Campbell et al., 2021) and negative self-appraisals (Pfeifer et al., 2013). Therefore, it is important to understand how peer environments are related to how adolescent girls think about themselves. This cross-sectional study sought to investigate how the quality of one’s friendships is related to self-judgements in adolescent girls aged 9–15 years, and how this relationship is associated with age.

The maturation of biological and cognitive processes, along with changes in adolescents’ social roles and environments, makes adolescence an important stage in the refinement of one’s self-concept: the multifaceted socio-cognitive construct encompassing knowledge and beliefs about the self (Pfeifer & Allen, 2021). Although ideas about selfhood emerge in early childhood (Cunningham et al., 2014; Harter, 2012), self-concepts become increasingly complex and abstract throughout adolescence. For example, young people’s descriptions of themselves become more domain-specific (e.g., academic, social, physical; Harter, 2012; Shapka & Khan, 2011; van der Cruijsen et al., 2023). In addition, the likelihood of adolescents endorsing negative self-judgements increases throughout early adolescence, peaking in mid-late adolescence (15–17 years) and decreasing in early adulthood (McArthur et al., 2019; van der Aar et al., 2018; van der Cruijsen et al., 2018). It has been proposed that cognitive and social processes associated with earlier pubertal development in girls when compared to boys are related to increased endorsement of negative self-appraisals in girls (Pfeifer & Allen, 2021; Pfeifer et al., 2013). In turn, the increase in negative self-evaluations during early and mid-adolescence in girls has been suggested to explain age and gender differences associated with poor mental-health and well-being outcomes, including internalising and externalising behaviours (Ybrandt, 2008), self-consciousness and social anxiety (Moses-Payne et al., 2022), and depressive symptoms (Bone et al., 2021; Shapka & Keating, 2005).

The development of social-cognitive processing abilities during adolescence might contribute to the rise of negative self-judgements in adolescent girls (Jankowski et al., 2014). For example, the continued development of the ability to ascertain the mental states of others (Dumontheil et al., 2010) allows adolescents to be more aware of the opinions of their peers, and subsequently reflect on how they think others perceive them (Crone & Steinbeis, 2017; Sebastian et al., 2008). In fact, this form of reflected self-concept becomes more central across adolescence. Studies show increased activation of brain regions that process social information during self-evaluation tasks (Güroğlu, 2022) and increasing similarities in self-evaluations in behavioural tasks using one’s own perspective and the perspective of others (Pfeifer et al., 2009; Van der Cruijsen et al., 2019; van der Cruijsen et al., 2023). Furthermore, increased awareness of the behaviours and abilities of peers has been related to an increased use of social comparisons to understand adolescents’ own behaviours and abilities (Crone et al., 2022; Pfeifer & Peake, 2012). In line with this, early adolescents internalise peer rejection, such that they view themselves more negatively after being rejected by peers (Rodman et al., 2017). In addition, one study found that girls (aged 9–13 years) engage in more self-critical social comparison processes, such as upward contrast (i.e., self-evaluation becoming more negative as a result of comparisons with someone judged as having better abilities; Gerber et al., 2018), compared to boys (Valls, 2022). Engaging in self-critical social comparisons is related to negative socioemotional responses (Noon & Meier, 2019; Pfeifer & Peake, 2012), including lower self-esteem (Orth et al., 2018; Rieger et al., 2016; Somerville et al., 2010) and higher self-consciousness (Rankin et al., 2004; Somerville, 2013; Somerville et al., 2013), particularly in early adolescence compared with other ages (Roeder et al., 2014).

Early adolescent friendships are marked by being relatively dynamic, with bonds between friends forming and breaking in short periods of time and becoming more stable across mid- and late adolescence (Burnett Heyes et al., 2015; Flannery & Smith, 2021; Stadtfeld et al., 2020). At the same time, sociocognitive development enables a myriad of relational behaviours to become more central in adolescent friendships compared to those of children, including reciprocity and loyalty, intimacy, self-disclosure, emotional support and conflict resolution (Flannery & Smith, 2017a; Güroğlu, 2022; Poulin & Chan, 2010). In turn, adolescents increasingly prefer to incorporate the views of close friends in their social decision-making (Slagter et al., 2023) and show similarities in self-evaluations and evaluations of close friends (van Buuren et al., 2020). Similarly, the preference to direct prosocial behaviour (e.g. in a resource allocation game) to friends rather than to socially distant peers increases with age during this period (Güroglu et al., 2014), particularly if these friendships are reciprocal (Burnett Heyes et al., 2015). Literature on the association between social relationships and self-cognitions in adolescence has typically focussed on the detrimental effect of peer victimisation on negative self-cognitions (e.g. Cole et al., 2016; Norrington, 2021; Roeder et al., 2014), as this is an important risk factor for developing mental health difficulties (Christina et al., 2021; Goemans et al., 2023; Roeder & Cole, 2018). In the current study, we focus instead on perceived friendship quality, which is a significant predictor of adolescent friendship stability and dissolution, psychosocial functioning, self-esteem and self-worth (Bagwell & Schmidt, 2011; Flannery & Smith, 2021; Harris & Orth, 2020), with high friendship quality being a protective factor against mental health problems (van Harmelen et al., 2021).

High friendship quality can be defined as a combination of high levels of positive interpersonal features, such as pro-social behaviour, self-disclosure, trust, intimacy and encouragement, alongside low levels of negative features, such as conflict and rivalry (Berndt, 2002). It has been proposed that friendship quality provides a space for the development of secure attachments, which are characterised by safety, trust and self-disclosure, and enable identity formation, positive self-esteem and self-worth (Gorrese & Ruggieri, 2013; Vijayakumar & Pfeifer, 2020). In addition, relationships characterised by low conflict promote feelings of social self-competence (Flannery & Smith, 2017a; Roeder et al., 2014) and are more likely to be maintained later in adolescence (Flannery & Smith, 2021). This suggests that there might be a bidirectional relationship between friendship and self-appraisals. For example, self-esteem and positive self-concepts are both linked to increased trust and self-disclosure in relationships (Tajmirriyahi & Ickes, 2020; Vijayakumar & Pfeifer, 2020). At the same time, conflict in peer relationships can lead to depressive symptoms and aversive behaviours, which in turn negatively impact relationships (e.g. interpersonal theory of depression; Rudolph, 2017; Stewart & Harkness, 2017).

The role of friendship quality on self-evaluation is likely to change as social and cognitive processes develop throughout adolescence. For example, heightened salience of socio-emotional contextual information (Albert & Newhouse, 2019; Goddings et al., 2012), coupled with relatively unstable self-concepts and social networks (Crone et al., 2022; Stadtfeld et al., 2020), could mean that the positive salience of good quality friendships plays a particularly important role in affective processes guiding self-evaluation in early adolescence (Rapee et al., 2019; Somerville, 2013). Conversely, as sociocognitive processes related to the recognition and attribution of mental states (e.g. social perspective taking) become more sophisticated (Crone & Steinbeis, 2017; Dumontheil et al., 2010; Flannery & Smith, 2017b; Van der Graaff et al., 2014), and quality of friendships becomes more stable and reciprocal (Burnett Heyes et al., 2015; Flannery & Smith, 2017a), it is possible that perceived friendship quality becomes a better predictor of adjustment and positive self-concepts later in adolescence.

In this cross-sectional study, we investigated the relationship between perceived friendship quality and self-judgements in adolescent girls and age-related differences in this relationship in early and mid-adolescence. We employed a self-appraisal task to measure the impact of positive and negative information on the self-judgements of girls between the ages of 9 and 15 years, compared to judgements of a chosen familiar other. This experimental task arguably provides a more sensitive measure of self-attributions than questionnaire-based measures of self-esteem and self-worth. In addition, we used a self-report questionnaire of friendship quality (van Harmelen et al., 2016), which was designed to measure both positive (e.g. trust, self-disclosure and closeness) and negative (e.g. conflict) experiences of close social relationships. We focused on girls as girls are more sensitive than boys to peer rejection (Joinson et al., 2012; Mendle et al., 2020) and tend to have more mental health problems in early adolescence than boys (Joinson et al., 2012). As peer relationships play an important role in the development of self-judgements, we firstly hypothesised that perceived friendship quality would be related to increased positive self-judgements (hypothesis 1a) and decreased negative self-judgements (hypothesis 1b) (both relative to judgements made about a chosen familiar other). In addition, given the biological, cognitive and socio-emotional development that occurs throughout the period of adolescence, we explored age-related differences in the strength of the relationship between perceived friendship quality and self-judgements (exploratory hypothesis 2).

## Methods

### Sample

This study used cross-sectional data collected between October 2019 and February 2020 for a larger study (Ahmed et al., 2024). The data from the larger study comprised 183 early adolescent girls between the ages of 9 and 15 years (M = 13.5, SD = 1.0), recruited from eight schools in London (see supplemental material SM1 for more details on school selection). Participants completed the study on individual computers in group sessions of between 2 and 26 pupils. In the current study, data from 20 participants were excluded due to technical difficulties during the self-appraisal task. Therefore, the final sample comprised data from 163 girls (aged 9–15 years, M = 13.1, SD = 1.0). Upon reviewer request, we have included an *a posteriori* power simulation in supplemental material SM1. The study was approved by the UCL Research Ethics Committee and carried out in accordance with General Data Protection Regulation (GDPR). Informed consent from parents and assent from all participants were obtained. Participants were compensated £10 in vouchers for taking part in a 1-h testing session. De-identified data and scripts are available on OSF at https://osf.io/dq9p3/.

### Procedure

We used a self-appraisal task to measure self-judgements, which was presented in Gorilla (https://gorilla.sc/), and the Cambridge Friendship Questionnaire (van Harmelen et al., 2016), which was presented in Qualtrics (https://qualtrics.com/), to measure perceived friendship quality. We also used the Pubertal Development Scale (Petersen et al., 1988) and an abbreviated version of the Raven’s Progressive Matrices test (Bilker et al., 2012) to allow adjustments for the potential confounding effects of differences in pubertal development and non-verbal reasoning abilities in this age range. Participants completed other cognitive tasks and questionnaires as part of the larger study, which are described elsewhere (Ahmed et al., 2024).

### Self-Appraisal Task

Self-judgements were measured using a task based on the self-appraisal paradigm described in Moses-Payne et al. (2022). In this task, participants were asked about positive or negative stimuli that referred to the participants themselves or another person. Participants chose a ‘familiar other’ whom they did not know personally (e.g. a celebrity or fictional character), as this has been suggested to offer greater differences in depth of processing (Sui & Humphreys, 2015; van Buuren et al., 2020). The 2 (word valence: positive, negative; within-subjects) x 2 (social condition: self, other; within-subjects) repeated measures design of the self-appraisal task is optimised to compare positive and negative judgements of self to those of a chosen familiar other (social control condition).

Participants had to rate how well a set of trait-descriptive adjectives described themselves (‘Does this word describe you?’) or the chosen other (‘Does this word describe [e.g., Hermione Granger]?’). Participants were given 7 s to judge the descriptiveness of the word from zero (‘Does not describe me/her at all’) to 10 (‘Totally describes me/her’). Participants judged a total of 64 words based on how well they described themselves (32 words: 18 positive, 14 negative) or their chosen familiar other (32 words: 18 positive, 14 negative). The order of the words was pseudo-randomised across social condition and word valence (see supplemental material SM2 for details about word allocation and randomisation). This task additionally included a memory component during which participants were asked whether they recognised the words presented in the task (described in Ahmed et al., 2024). We did not analyse this data here as the current analysis focused on self-evaluation (and not memory).

Descriptiveness ratings of positive and negative trait-descriptive adjectives for oneself provided during the task were used as measures of positive self-judgements and negative self-judgements. Self-judgements therefore refer to the extent to which an individual believes they are accurately represented by trait-descriptive adjectives (Moses-Payne et al., 2022). Negative and positive judgements about the chosen other were measured in the same way.

### Friendship Quality Questionnaire

Perceived friendship quality was measured using the Cambridge Friendship Questionnaire (van Harmelen et al., 2016), an 8-item self-report questionnaire derived from a semi-structured interview on social relationships (Goodyer et al., 1989). This questionnaire asks participants to rate the extent to which they agree with a set of indicators of closeness of friendships and conflict behaviours (e.g., ‘How often do you see your friends outside of school?’, ‘Do you have arguments with your friends that upset you?’) on a four or six-point Likert scale. This questionnaire does not measure structural components of friendships, such as the number of friends in the participant’s network. Items are added to produce a score ranging from zero to 32, with higher scores indicating greater perceived friendship quality. The questionnaire has good measurement invariance and external validity (van Harmelen et al., 2016).

### Pubertal Development Scale

To enable adjustment for the potential confounds of pubertal development, participants completed the Pubertal Development Scale (PDS; Petersen et al., 1988). This is a self-report scale that assesses five general indicators of development (growth in height, skin changes, growth of body, breast development and menarche), for example: “Have your breasts begun to grow? 1 = no, 2 = yes barely, 3 = yes definitely, 4 = development completed”. The scores of these five questions were averaged to give a continuous score ranging from 1 (pre-pubertal) to 4 (completed pubertal development). The question about onset of menarche was rated on a 3-point scale (1 = no, 2 = yes barely & 3 = yes definitely) but was recoded so that ‘no’ was scored as 1 and both ‘yes barely’ and ‘yes definitely’ were scored as 4 (in line with previous work; see Carskadon & Acebo, 1993). Pubertal stage of the participants ranged from 1.4 to 3.8 (M = 2.8, SE = .6) and no participant reported complete pubertal maturation (a score of 4).

### Non-Verbal Reasoning

To enable adjustment for the potential confounds of non-verbal reasoning, participants completed a nine-item abbreviated version of the Raven Standard Progressive Matrices Test, which is related to IQ (Bilker et al., 2012) The number of correct answers was summed to give a continuous score from zero to 9. Non-verbal reasoning scores of the participants ranged from 1.0 to 9.0 (M = 6.0, SE = 1.7). One participant did not have non-verbal reasoning data due to computer issues.

### Statistical Analysis

The dependent variable was participant *judgements*, measured by taking each descriptiveness rating for the trait-adjectives presented in the self-appraisal task. The *social condition* and *word valence* factors of the self-appraisal task were used as independent variables, as well as *friendship quality* (as determined by the sum of the friendship quality questionnaire) and participant *age*. Both *friendship quality* and *age* were modelled as continuous linear variables and were mean-centred to meet assumptions of multicollinearity. We used decimal *age* (rounded to the nearest 100th).

Raw trial-level *judgements* were modelled using a linear mixed effects model (*lmerTest* package version 3.1–3; Kuznetsova et al., 2017) in the R programming environment (R version 4.3.2; R Core Team, 2023). To test whether perceived friendship quality cross-sectionally predicts differences in self-judgements (H1), the first model regressed *judgements* on the three-way interaction between *friendship quality*, *social condition* and *word valence*, as well as their two-level interactions and main effects. To test whether the relationship between perceived friendship quality and self-judgements differed cross-sectionally with age (EH2), the second model additionally included *age* (and all possible three-way interactions among this and the other variables of the model). To obtain more parsimonious models, we progressively excluded nonsignificant higher-level interactions via nested model comparison. Both models clustered data by participant (as a random intercept) and included random slopes for within-subject factors as random effects (*social condition* and *word valence*). Main effects and interactions of the best-fitting model were inspected using Type III Wald F-tests with Satterthwaite approximations for degrees of freedom. We converted F values of significant main effects and interactions to estimated effect sizes of η_p_^2^ (partial eta-squared; confidence interval = 95%) using the *effectsize* package (version 0.8.6; Ben-Shachar et al., 2020). Post hoc comparisons were performed using the *emmeans* package (version 1.8.5; Lenth et al., 2022) and were Bonferroni-corrected for two comparisons (*social condition* and *word valence*).

We tested the robustness of the main effects and interactions by running a set of sensitivity analyses including additional covariates to the models described above, which we refer to here as control models. To control for differences in pubertal development in this age group, which is related to sensitivity to social-emotional processing (Pfeifer & Peake, 2012), the first control model additionally included pubertal development as a covariate (CM1). The second control model additionally included non-verbal abilities as a covariate (CM2). We additionally controlled for testing group size (i.e. number of participants taking part in the same testing session, CM3) as peer presence could affect social information processing (Breiner et al., 2018). Finally, we controlled for single gender schools (CM4). Pubertal development, non-verbal reasoning and testing group size were all modelled as continuous linear variables, whereas single gender school was modelled as a binary factor (single gender, co-education). See supplemental material SM3 for full details about fixed- and random-effect structure of all models. Data and scripts are available on OSF at https://osf.io/dq9p3/.

## Results

**Figure 1.**
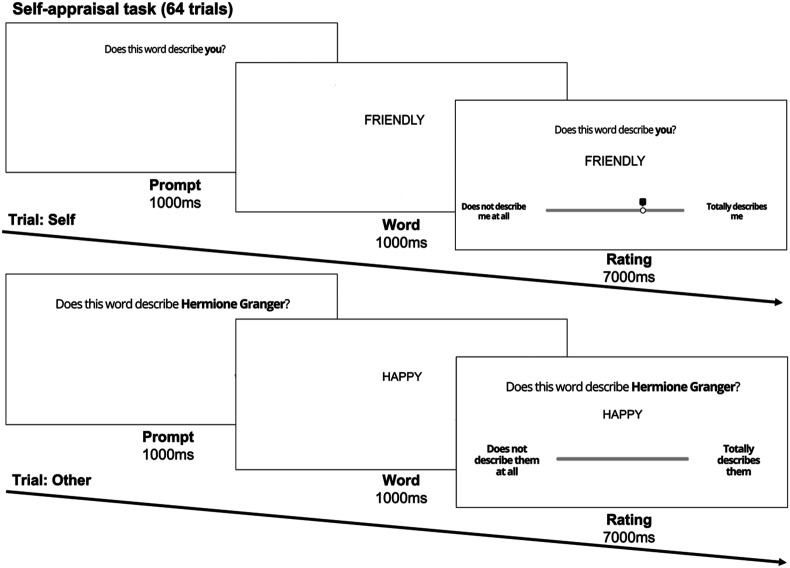
Self-appraisal task. Participants rated whether a set of 64 words were descriptive of themselves (32 words: 18 positive, 14 negative) or of a chosen familiar other (e.g., Hermione Granger; 32 words: 18 positive, 14 negative) on a scale from zero (Does not describe me/them at all) to 10 (Totally describes me/them). Participants had 7 s to provide their rating. Words were pseudo-randomised (see supplemental material SM1 for details).

Overall, descriptive statistics of the self-appraisal task replicated previous findings relating to mean self-appraisal and appraisals of a chosen other in this age group (self-judgements: M_positive_ = 6.0, SD_positive_ = 2.7, M_negative_ = 3.6, SD_negative_ = 2.9; other judgements: M_positive_ = 6.3, SD_positive_ = 2.9, M_negative_ = 3.0, SD_negative_ = 3.0; Moses-Payne et al., 2022; van der Cuijsen et al., 2018; van der Aar et al., 2018, see Figure 1). Self-reported friendship quality was comparable to the previous literature using this scale (M = 24.0, SD = 4.0, IQR = 22 - 27; van van Harmelen et al., 2016; van Harmelen et al., 2017; van Harmelen et al., 2021) and was not correlated with age (r = −.04, *p* = .617).

### Hypothesis 1. Effect of Perceived Friendship Quality on Judgements

**Figure 2.**
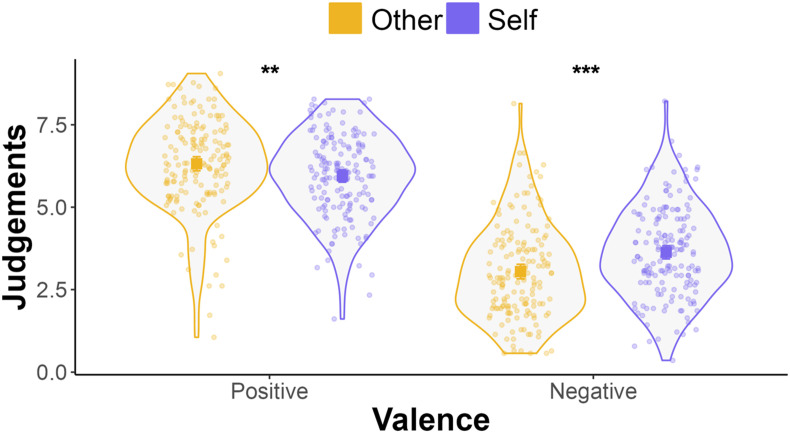
Interaction between word valence and social condition on judgements. The violin plots represent kernel probability density of judgements (0 – 10; descriptiveness ratings from the self-appraisal task) grouped by word valence (positive on the left and negative on the right) and social condition (self-judgements in purple and judgements of the chosen other in orange). Dots represents participant-level mean judgements. Squares show group mean judgements as estimated by the linear mixed effects model and error bars show the 95% confidence intervals (negligible). Asterisks represent *** *p*_Bonf_ < .001, ** *p*_Bonf_ < .01.

The results of the linear mixed effects model showed a main effect of word valence (*F* (1,161) = 411.61, *p* < .001; *η*_*p*_^*2*^ *=* .72, CI [.65 .77]), such that positive judgements were higher than negative judgements, which has been referred to as a positivity effect (contrast _negative - positive_ = −2.79, SE = .14, *p* < .001; Moses-Payne et al., 2022). There was no main effect of social condition (*F* (1,161) = 2.41, *p* = .123). The association between social condition and judgements was dependent on word valence: there was a two-way interaction between social condition and word valence (*F* (1,161) = 19.82, *p* < .001; *η*_*p*_^*2*^ *=* .11, CI [.04 .21]), which was driven by higher negative self-judgements than negative judgements about the chosen other (contrast _other - self_ = −.58, SE = .13, *p*_Bonf_ < .001), and lower positive self-judgements than positive judgements about the chosen other (contrast _other - self_ = .37, SE = .13, *p*_Bonf_ = .007, see Figure 2).

**Figure 3.**
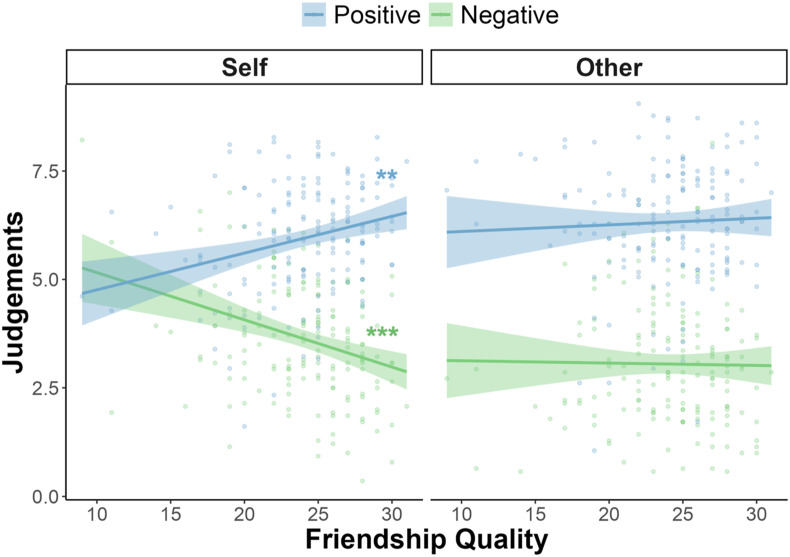
Relationship between perceived friendship quality and judgements depends on social condition (self in the left; other in the right) and word valence (positive in blue; negative in green). The plot shows judgements (0 – 10; descriptiveness ratings from the self-appraisal task) as a function of participant friendship quality (0 – 32), social condition (self or chosen other) and word valence (positive or negative). Data points are participant-level mean judgements. A linear mixed effects model showed that mean positive self-judgements (blue line) increased with perceived friendship quality and mean negative self-judgements (green line) decreased with perceived friendship quality (left panel). The right panel shows that positive (blue line) and negative judgements (green line) for the chosen other did not change significantly with perceived friendship quality (right panel). Shaded areas represent 95% confidence intervals. Asterisks show Bonferroni corrected *p*-values: ^***^p_Bonf_ < .001; ^**^p_Bonf_ < .01.

There was no main effect of perceived friendship quality (*F* (1,161) = 0.12, *p* = .729). Instead, there was a two-way interaction between perceived friendship quality and word valence (*F* (1,161) = 10.43, *p* = .002; *η*_*p*_^*2*^ *=* .06, CI [.01 .14]): higher perceived friendship quality was related to increased positive judgements (slope = .05, SE = .02, *p*_*Bonf*_ = .020) and decreased negative judgements (slope = −.06, SE = .02, *p*_*Bonf*_ = .012). In addition, there was a significant three-way interaction between perceived friendship quality, word valence and social condition (*F* (1,161) = 10.59, *p* = .001; *η*_*p*_^*2*^ *=* .06, CI [.01 .15]; see Figure 3). In support of hypothesis 1, simple effects showed that higher friendship quality predicted higher positive self-judgements (slope = .09, SE = .02, *p*_Bonf_ = .001; Figure 3, left panel, blue line) as well as lower negative self-judgements (slope = −.11, SE = .03, *p*_Bonf_ < .001; Figure 3, left panel, green line). Positive judgements for the chosen other (slope = .02, SE = .03, *p*_Bonf_ = 1; Figure 3, right panel, blue line) and negative judgements for the chosen other (slope = −.01, SE = .03, *p*_Bonf_ = 1; Figure 3, right panel, green line) were not significantly different across perceived friendship quality. Post-hoc comparisons showed that the difference between negative self-judgements and negative judgements for the chosen other become smaller with increasing perceived friendship quality (contrast _other - self_ = .11, SE = .03, *p*_Bonf_ = .002). The difference between positive self-judgements and positive judgements for the chosen other followed a similar trend but was not statistically significantly (contrast _other - self_ = −.07, SE = .03, *p*_Bonf_ = .060). These effects were robust to all sensitivity analyses (see supplemental material SM4).

### Exploratory Hypothesis 2: Effect of age on the relationship between Perceived Friendship Quality and Judgements

**Figure 4.**
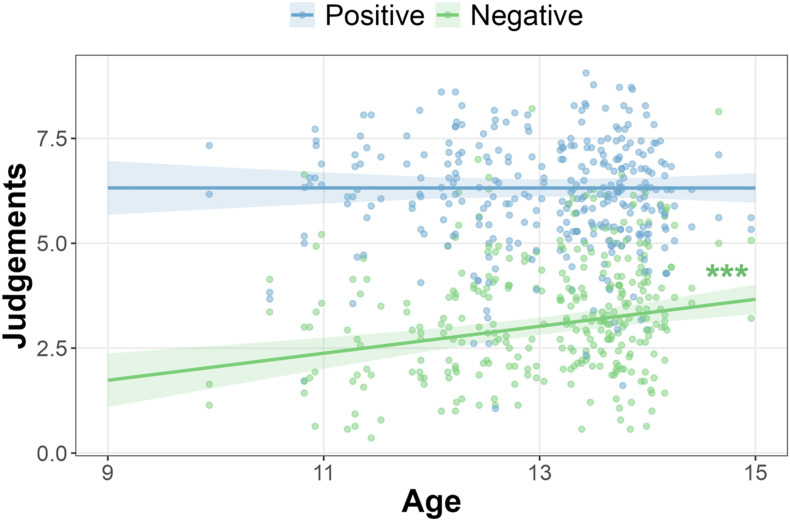
Positivity effect (higher positive compared to negative judgements) decreases with age. The plot shows mean judgements (0 – 10; average computed from trait-descriptive adjectives rated during the self-appraisal task) as a function of participant age and word valence (positive or negative). Data points are participant-level mean judgements. A linear model showed that mean negative judgements increased with age (green line), while mean positive judgements did not change significantly with age (blue line). Shaded areas represent 95% confidence intervals). Asterisks show Bonferroni corrected *p*-value: ^***^p_Bonf_ < .001.

In addition to the terms described above, a second linear mixed effects model additionally showed a main effect of age (*F* (1,160) = 11.33, *p* < .001; *η*_*p*_^2^ = .07, CI [.01 .15]) and a two-way interaction between age and word valence (*F* (1,160) = 5.63, *p* = .019; *η*_*p*_^2^ = .03, CI [0 .11]; see Figure 4). While higher judgements overall in the self-appraisal task were associated with older age (slope = .31, SE = .08, *p* < .001), simple effects showed that this effect was driven by higher negative judgements in the self-appraisal task being associated with older age (slope = .32, SE = .09, *p*_Bonf_ < .001). This was not true for positive judgements, which did not differ significantly as a function of age (slope = 0, SE = .08, *p*_Bonf_ = 1). This resulted in a smaller positivity effect associated with older age, whereby the difference between positive judgements and negative judgements was smaller at older ages (contrast _negative - positive_ = .33, SE = .14, *p_Bonf_* = .035, see Figure 4). As age and word valence did not further interact with the social condition of the self-appraisal task (i.e., there was no three-way interaction), the results suggest that the age-related increase in negative judgements was not specific to self-judgements, but rather general across both self-judgements and judgements for the chosen other. These effects were robust to all sensitivity analyses (see supplemental material SM4). Notably, a model including higher-level interactions of age and friendship quality did not fit the data better than this model (*χ*^2^ (6) = 2.16, *p* = .904). This means that there is not sufficient evidence to suggest that there were age-related differences in the relationship between friendship quality and judgements in our sample.

## Discussion

This study used a self-appraisal task and self-reported friendship quality to cross-sectionally investigate the relationship between perceived friendship quality and self-judgements in adolescent girls aged 9–15 years. We found that higher self-reported friendship quality was related to lower negative self-judgements and higher positive self-judgements. In contrast, this relationship was not found for judgements of a chosen familiar other. In addition, we did not find evidence for an age-related difference in the relationship between perceived friendship quality and self-judgements. However, we found that negative judgements in the self-appraisal task, but not positive judgements, were associated with older age, suggesting that a positivity effect in judgements, whereby positive judgements are higher than negative judgements, was smaller in older adolescents.

### The Relationship between Perceived Friendship Quality and Positive and Negative Self-Judgements

In support of our first hypothesis, we found that higher self-reported friendship quality was related to higher positive self-judgements and lower negative self-judgements. This is in line with previous work suggesting that the quality of interpersonal relationships during adolescence is related to increased well-being and psychosocial functioning (Alsarrani et al., 2022; van Harmelen et al., 2017), and reduced feelings of anxiety and depression (van Harmelen et al., 2016), particularly in girls (Rueger et al., 2010). Self-esteem has been suggested to be an important factor for how social experiences are integrated into the self-concept (Crone et al., 2022), and therefore, one mechanism by which perceived friendship quality is related to positive self-concepts might be increased feelings of self-worth and self-esteem (Harris & Orth, 2020; Maunder & Monks, 2019). For example, friendship quality is defined by experiencing security, closeness and intimacy in friendships (Bagwell & Schmidt, 2011), and could be related to self-esteem through increased feelings of social acceptance and belonging (Tomova et al., 2021). In addition, self-esteem could be influenced by direct positive feedback from friends (e.g., validation and compliments) and fewer negative social experiences, such as receiving hurtful criticism or being victimised (Bagwell & Schmidt, 2011; Duru et al., 2019; Güroğlu, 2022; van Harmelen et al., 2021). Given that conflict in peer relationships and victimisation are consistently related to depressive cognitions (e.g. Cole et al., 2016; Norrington, 2021), and low self-esteem is a predictor of mental health difficulties (Rieger et al., 2016), understanding how friendship quality is related to self-esteem and positive self-cognitions could be a fruitful avenue for understanding links between self-processing and mental well-being.

Another explanation is that having good quality friendships might reduce negative biases in self-evaluative processing when compared to evaluative processing of others. Good quality friendships could be related to perceived similarity with close friends, which might be beneficial for self-evaluation (van Buuren et al., 2020). Research on social comparisons has suggested that comparing oneself to similar others has a greater influence on self-esteem and self-judgements than comparing ourselves to more socially distant individuals (e.g., Tarrant et al., 2006). In addition, literature on the self-enhancement model in young adults suggests that evaluations between the self and others are more similar in high quality relationships (Morry & Sucharyna, 2019), and that people are motivated to include close friends in self-concepts to expand the self (e.g., Aron et al., 2022; Gardner et al., 2002). Friendship quality has also been linked to increased social perspective taking in mid- and late adolescence (Flannery & Smith, 2017b; Rokeach & Wiener, 2022), which could be related to the use of similar strategies to make self-judgements and judgements about other people. In line with this, our results show that negative self-judgements and negative judgments of chosen others become more similar with higher perceived friendship quality. In fact, social network research suggests that stable networks are characterised by peer similarity and shared characteristics (Stadtfeld et al., 2020), which could mean that people choose to associate with individuals who are similar to them (Kandel, 2017). This contributes to a bidirectional relationship between the formation of stable social networks and the role of social comparisons in self-evaluations. Note, however, that our self-appraisal task uses a socially distant familiar other as a control social condition, as this has been suggested to offer greater differences in depth of processing (Sui & Humphreys, 2015; van Buuren et al., 2020). Future research could investigate the relationship between friendship quality and self-judgements compared to those of socially close others (e.g. a best friend). It is important to acknowledge that favourable opinions of friends and social desirability bias might influence judgements to be at ceiling (positive) or at floor (negative). In sum, further studies could investigate the role of perceived similarity in friendship groups, and how this is related to different strategies employed to make judgements about the self, friends and larger peer groups.

### Age-Related Differences in the Relationship between Perceived Friendship Quality and Self-Judgements

In general, our results are consistent with previous literature on age-related differences in self-judgements during adolescence in girls: negative self-judgements increase during adolescence, but positive self-judgements remain relatively stable in cross-sectional and longitudinal studies (Moses-Payne et al., 2022; van der Aar et al., 2018; van der Cruijsen et al., 2018). This was true for both self-judgements and judgements about the chosen others. This suggests that the positivity effect consistently found in evaluations in children, young people and adults (Beer, 2014; Moses-Payne et al., 2022), whereby we view ourselves and others more positively than negatively, might be less pronounced during early and mid-adolescence. It has been suggested that self-concepts transition away from a positivity effect in the process of general self-evaluations becoming more domain-specific (Crone et al., 2022). For example, the onset of puberty has been suggested to mark a turning point in the neurocognitive mechanisms that make young people more sensitive to their social-emotional context during self-evaluation (Barendse et al., 2020; Pfeifer & Peake, 2012). In line with this, young people might use different strategies to inform self-concepts across development: while children might rely on parental relationships for informing self-judgements, adolescents might rely on using information from their social environment, and potentially shift to rely more on self-relevant memory in adulthood (Cunningham & Turk, 2017; Pfeifer et al., 2009; Yoon et al., 2018). Our results show that a decrease in positivity effect across adolescence remains after taking pubertal development into account, which suggests that sociocultural contexts and structures might also play a role in forming evaluative self-judgements across early and mid-adolescence.

We hypothesised that there would be an age-related difference in the relationship between perceived friendship quality and self-judgements throughout early to mid-adolescence. It is possible that having good quality friendships would be particularly important for self-evaluation during early adolescence, when self-concepts are more unstable and potentially more sensitive to contextual influences. In contrast, another possibility was that an improved ability to engage in reflected self-appraisals and social comparisons with good friends during mid-adolescence would be more significant for self-evaluation during that period. However, our results suggested that the relationship between perceived friendship quality and self-judgements did not differ between early and mid-adolescence. There are at least two explanations for these null findings. On the one hand, friendship quality increases with age throughout adolescence (Poulin & Chan, 2010; van Harmelen et al., 2021), and, therefore, the age range of our sample (9–15 years) might not be large enough to observe a difference in the relationship between perceived friendship quality and self-judgements. In addition, our self-report friendship quality questionnaire might not have been sensitive enough to detect nuances in good quality friendships. For example, differences in trust, intimacy and communication, or factors related to structure of networks, such as quantity and stability of peer relationships, might be independently related to self-evaluation processes in early and mid-adolescence (Barzeva et al., 2022). In fact, the self-reported friendship quality questionnaire does not consider whether a young person might have no friends. Future studies could develop methods to measure greater nuances in the quality of friendships and peer relationships, leading to work showing how these might individually predict self-evaluations in early, mid and late adolescence.

### Limitations and Future Research Directions

The findings of this study should be interpreted considering certain conceptual and methodological limitations. First, as has been discussed throughout the paper, due to the cross-sectional nature of our data, our results cannot discern any causal or longitudinal relationship between perceived friendship quality and self-judgements. An alternative interpretation of our results is that the girls in our sample with positive self-concepts are better able to maintain high quality friendships. In fact, self-esteem and positive self-concept have been linked to increased self-disclosure (Tajmirriyahi & Ickes, 2020; Vijayakumar & Pfeifer, 2020), the action of sharing personal information about oneself with others, which is thought to be a component of friendship quality (Cuadros & Berger, 2022; Towner et al., 2022). In addition, low conflict in high quality friendships is related to increased social self-competence and use of conflict resolution strategies (Flannery & Smith, 2017a, 2021). Therefore, the link between friendship quality and self-evaluation is likely to be complex and bidirectional, possibly reflecting a feedback loop over time (Harris & Orth, 2020). We were unable to explore this dynamic relationship using data from a second timepoint, given that the data collection of the larger study was interrupted by the outbreak of the coronavirus pandemic in 2020, and the friendship quality measure in the second timepoint was heavily confounded by social distancing restrictions imposed by the pandemic (e.g., questionnaire items asking participants to report how many times they see their friends). This also resulted in data collection being halted for a group of 9- and 15-year-old girls, which means that our findings might be most generalisable to girls between 10 and 14 years of age. Longitudinal studies across early and mid-adolescence should be conducted to understand how friendship group dynamics are important for self-development, as well as how particular self-perceptions could be related to how young people navigate their social environments.

Finally, there were practical limitations associated with using data from a larger study. First, the larger study only included data from girls. There are several reasons why we might expect to find gender differences in the relationship between friendship quality and self-judgements across adolescence: girls tend to have greater negative self-concepts (van der Cruijsen et al., 2018) and mental health problems (Bone et al., 2021), have an earlier onset of puberty on average (Dorn, 2006) and show higher neural sensitivity to socioemotional information (Pfeifer et al., 2013). In addition, girls tend to show, on average, greater social perspective taking and empathic concern in close relationships (Flannery & Smith, 2017b), and these gender differences are exacerbated in environments that adopt more traditional gender roles (Van der Graaff et al., 2014). Notably, single gender schools have been suggested to challenge traditional gender expectations and increase mathematics and science-related self-beliefs in girls (see review in Robinson et al., 2021). This highlights the potential in considering the school context in adolescent friendship dynamics. Second, while we explored age-related differences in the relationship between friendship quality and self-appraisals, the larger study did not include other factors that could potentially modify this relationship. For example, girls from poorer households and from ethnic minorities are at greater risk of experiencing mental health difficulties (Patalay & Fitzsimons, 2017), which might be associated with unique social pressures and negative self-appraisals (e.g. Deardorff et al., 2019). The larger study prioritised recruitment from schools that had continuing education from primary school and secondary school, which resulted in the majority of schools being independently funded, and therefore our sample is not likely to be generalisable to the UK population. Taken together, future studies are needed to understand whether our results are generalisable to young people who have different experiences related to their identity or socioeconomic background. In addition, given that the larger study was not designed for the analyses employed in this study, future studies should replicate these findings using a study design optimised for detecting meaningful effects using mixed effects models.

## Conclusion

The current study suggests that the quality of friendships could be an important component in self-evaluations in early- and mid-adolescence in girls. Understanding the mechanisms underlying the relationship between friendship quality and positive self-judgements - such as positive feedback, increased perceived similarity with close friends, and reductions of cognitive biases - could inform current literature exploring self-concept training as a tool for integrating positive experiences into one’s self-concept (Van der Aar et al., 2022). In addition, we found a decrease in positivity effect from early adolescence and into mid-adolescence (Beer, 2014), which could be related to changes in the neurocognitive strategies underlying heightened negative self-appraisals during adolescence, such as the use of peer comparisons (Crone et al., 2022). Future studies should investigate how larger social and cultural structures (e.g., social inequalities and social media use) can amplify vulnerabilities related to neurocognitive development and mental health in young people (Choudhury et al., 2023).

## Supplemental Material

Supplemental Material - The Relationship Between Perceived Friendship Quality and Self-Judgements in Adolescent Girls from London Supplemental Material for The Relationship Between Perceived Friendship Quality and Self-Judgements in Adolescent Girls from London by Blanca Piera Pi-Sunyer, Jessica Evans, Katy Ratcliffe, Kaushalya Janaarthanan, Saz Ahmed, Willem Kuyken, Tim Dalgleish, and Sarah-Jayne Blakemore in The Journal of Early Adolescence

## Data Availability

The analysis scripts and data used in this study can be found in the Open Science Framework (https://osf.io/dq9p3/).
