# Supplementary material for: The Relationship between Perceived Friendship Quality and Self-Judgements in Adolescent Girls from London
Source: J Early Adolesc. 2024 Aug 5;45(9):1183–211. doi: 10.1177/02724316241271327 (PMC12443375; doi:10.1177/02724316241271327)
Supplement: Supplemental Material - The Relationship Between Perceived Friendship Quality and Self-Judgements in Adolescent Girls from London [file sj-pdf-1-jea-10.1177_02724316241271327.pdf]

Supplemental Material

SM1. Sample characteristics

School selection

Participants were recruited from eight schools in London. To recruit children across the entire age range studied (9-15 years) from within the same settings, we prioritised recruitment from schools that had continuing education from primary school (reception to Year 6; ages 4 to 11) to secondary school (Year 7 to Year 11 or 13; ages 11 to 16 or 18). The recruited schools comprised one state-funded and four independently funded (fee paying) single gender schools, and three independently funded co-education schools.

A posteriori power simulation

The sample size was determined by a power analysis designed for a mediation analysis conducted in the original study (Ahmed et al., 2024). Upon reviewer request, we ran a power simulation of the model estimates reported in the manuscript varying by 100, 150 and 200 participants, using the *mixedpower* package and the methodology proposed by Kumle and colleagues, (2021). The simulation suggests that a sample of 150-200 participants has ≥79% power to detect the main effects and interactions reported for the analysis 1 mixed effects model (see Figure S1). We did not run a power simulation for the analysis 2 mixed effects model as this analysis tests an exploratory hypothesis.

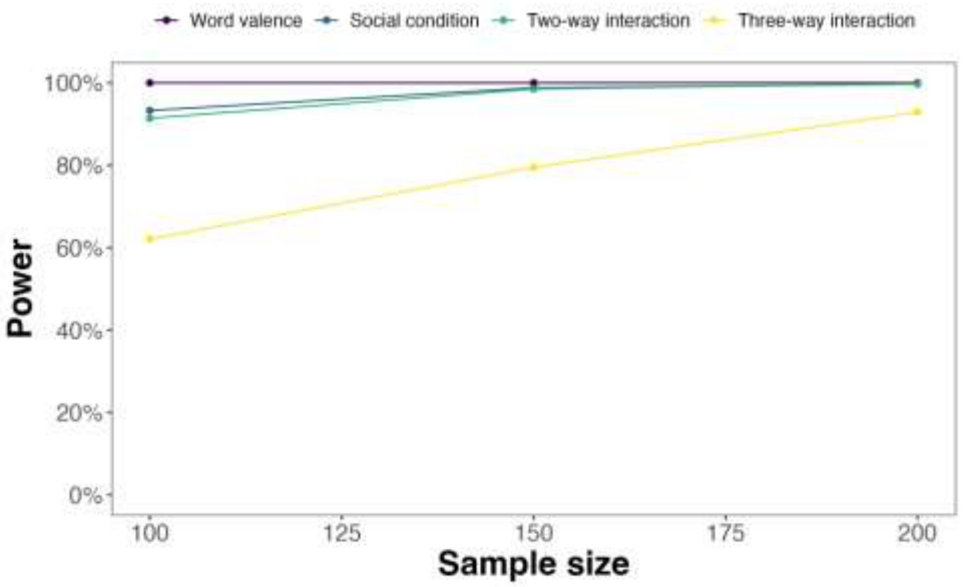

Figure S1. A posteriori power simulation. The figure shows a power simulation for the model estimates reported in the manuscript varying by sample size (100, 150, 200). The lines show that the main effects of word valence (purple line) and social condition (blue line), and the two-way interaction between social condition and word valence (green line) are detected >80% power at all sample size simulations. The three-way interaction between social condition, word valence and friendship quality is detected at >79% power at sample sizes greater than 150 participants.

SM2. Allocation of words for self-appraisal task

Words were generated using the R package LexOPS (Taylor et al., 2020). Valence ratings were taken from Warriner and colleagues (2013) and a median split of the ratings were used to categorise words as positive or negative. Words had an average age of acquisition of 6.53 (SD = 1.46) and all words had an age of acquisition below the age of 8 (age of acquisition taken from Brysbaert & Biemiller, 2017).

The allocation of words to either target or distractor was pseudo-randomised across participants. To this end, 128 words were split into eight lists of 16 words (see Table S1). For

each participant, an algorithm randomly selected four lists to be displayed during the task (two lists for self-judgements, two lists for judgements for the chosen other). Words were displayed sequentially in a random order.

Table S1

Word	Valence rating (Warriner et al., 2013)	Positive (1) vs Negative (0)	Age of acquisition (Brysbaert & Biemiller, 2017)	Randomisation List
**jealous**	2.38	0	7.42	1
**unfriendly**	2.3	0	7.00	1
**boring**	2.71	0	5.26	1
**forgetful**	3.59	0	7.84	1
**liar**	2.41	0	4.89	1
**friendless**	3.15	0	7.95	1
**sassy**	5.19	0	8.22	1
**quiet**	6.47	1	4.15	1
**peaceful**	8	1	7.22	1
**funny**	7.59	1	5.47	1
**attractive**	7.19	1	8.58	1
**calm**	6.89	1	6.26	1
**clever**	7.36	1	7.50	1
**fair**	7.14	1	5.67	1
**heroic**	7.45	1	8.20	1
**religious**	5.2	1	7.84	1
**clumsy**	4.14	0	7.26	2
**lazy**	3.05	0	6.39	2
**cruel**	2.73	0	7.63	2
**lonely**	2.67	0	5.80	2
**unkind**	2.55	0	7.21	2
**fussy**	2.71	0	6.67	2
**scary**	3	0	4.26	2
**patient**	6.71	1	7.05	2
**scientific**	5.95	1	8.74	2
**helpful**	7.43	1	5.44	2
**strong**	6.81	1	4.58	2
**popular**	5.71	1	6.61	2
**understanding**	7.14	1	7.83	2
**famous**	5.89	1	6.84	2
**lovable**	8.26	1	7.68	2
**brilliant**	7.5	1	7.95	2
**unlucky**	2.7	0	7.05	3
**unpopular**	2.95	0	8.20	3

**nosey**	3.1	0	5.47	3
**shy**	5.16	0	5.32	3
**impolite**	3.1	0	7.26	3
**grumpy**	2.81	0	5.89	3
**scruffy**	4.42	0	8.24	3
**talented**	7.95	1	8.25	3
**relaxed**	7.25	1	7.63	3
**athletic**	6.76	1	8.55	3
**polite**	6.57	1	4.63	3
**sensible**	6.74	1	8.78	3
**beautiful**	7.61	1	5.72	3
**gentle**	7.42	1	4.89	3
**lovely**	7.55	1	7.26	3
**silly**	6.72	1	4.17	3
**weak**	2.95	0	5.58	4
**sad**	2.1	0	3.24	4
**unreliable**	2.74	0	8.89	4
**chatty**	4.42	0	8.22	4
**bossy**	2.86	0	7.06	4
**horrible**	2.33	0	5.81	4
**sneaky**	3.94	0	6.58	4
**brave**	7.38	1	6.00	4
**smart**	7.73	1	5.50	4
**cautious**	6.05	1	8.25	4
**imaginative**	6.65	1	8.74	4
**friendly**	7.84	1	4.50	4
**charming**	7.05	1	6.26	4
**girly**	5.32	1	6.81	4
**lucky**	7.32	1	5.89	4
**special**	7.5	1	5.00	4
**greedy**	2.1	0	7.53	5
**untidy**	3.58	0	8.72	5
**unhealthy**	2.55	0	6.78	5
**childish**	4.89	0	6.33	5
**irritating**	2.63	0	8.53	5
**horrid**	2.68	0	8.50	5
**strange**	4.72	0	6.42	5
**musical**	7.68	1	7.28	5
**happy**	8.47	1	2.72	5
**tidy**	6.24	1	8.53	5
**cheerful**	8	1	7.68	5
**likable**	6.83	1	7.00	5
**clean**	7.09	1	3.89	5
**good**	7.89	1	3.55	5
**naughty**	6.04	1	4.90	5
**sporty**	5.78	1	7.05	5

**annoying**	3	0	6.52	6
**careless**	3.53	0	8.37	6
**foolish**	3	0	8.94	6
**messy**	3	0	5.05	6
**dorky**	5.12	0	6.90	6
**nasty**	2.95	0	6.00	6
**tough**	4.45	0	5.47	6
**energetic**	7.57	1	6.90	6
**nice**	6.95	1	3.95	6
**lively**	7.12	1	8.60	6
**trustworthy**	7.25	1	8.05	6
**proud**	7	1	5.44	6
**cool**	6.82	1	4.58	6
**goofy**	6.71	1	4.83	6
**perfect**	7.19	1	5.78	6
**stylish**	5.68	1	8.39	6
**dishonest**	3	0	5.68	7
**noisy**	3.21	0	5.33	7
**selfish**	3.3	0	5.26	7
**impatient**	3.53	0	7.39	7
**dumb**	2.44	0	4.50	7
**odd**	4.56	0	6.00	7
**ugly**	2.47	0	4.17	7
**neat**	6.95	1	5.43	7
**wise**	7.42	1	6.79	7
**intelligent**	7.6	1	8.28	7
**interesting**	6.78	1	6.95	7
**serious**	5.88	1	6.58	7
**cute**	7.56	1	4.26	7
**gorgeous**	7.57	1	7.68	7
**positive**	7.57	1	8.11	7
**sweet**	7.77	1	4.53	7
**rude**	2.04	0	7.11	8
**unhappy**	1.84	0	4.89	8
**stubborn**	3.74	0	7.17	8
**stupid**	2.84	0	4.40	8
**evil**	2.34	0	6.71	8
**picky**	3.9	0	5.74	8
**weird**	5.09	0	5.61	8
**kind**	7.78	1	4.89	8
**creative**	7.06	1	8.74	8
**honest**	8.16	1	5.46	8
**leader**	6.24	1	6.90	8
**generous**	7.43	1	7.22	8
**fabulous**	7.84	1	7.83	8
**healthy**	7.76	1	7.61	8

**powerful**	6.46	1	6.42	8
**wonderful**	7.41	1	6.47	8

Self-appraisal task – valence ratings, age of acquisition, and randomisation of words

SM3. Model Specifications

For each model described in the manuscript, we report here its full random-effects structure and fixed effect estimates.

	**Dependent Variable**	**Fixed Effects**	**Random Effects**
**Model 1**	Trial-level judgements	Friendship Quality, Valence, Person (interaction)	Valence, Person (interaction)
**Model 2**	Trial-level judgements	Friendship Quality, Valence, Person (interaction),<br>Valence, Age (interaction)	Valence, Person (interaction)
**Control Model 1**	Trial-level judgements	Friendship Quality, Valence, Person (interaction),<br>Valence, Age (interaction),<br>Pubertal Development	Valence, Person (interaction)
**Control Model 2**	Trial-level judgements	Friendship Quality, Valence, Person (interaction),<br>Valence, Age (interaction),<br>Non-verbal Reasoning	Valence, Person (interaction)
**Control Model 3**	Trial-level judgements	Friendship Quality, Valence, Person (interaction),<br>Valence, Age (interaction),<br>Testing group size	Valence, Person (interaction)
**Control Model 4**	Trial-level judgements	Friendship Quality, Valence, Person (interaction),<br>Valence, Age (interaction),<br>Single gender school	Valence, Person (interaction)

Table S2

Model Specifications

SM4. Sensitivity Analyses

We tested the robustness of the main effects and interactions by running a set of sensitivity analyses including additional covariates to the models described in the manuscript. All main effects and interactions were robust to the inclusion of pubertal development, non-verbal reasoning, testing group size and single gender schools. Testing group size and single gender schools were the only covariates that had additional main effects on judgements in the self-appraisal task ($F(1,159)_{\text{Testing group size}} = 6.18$, $p = .014$; $\eta_p^2 = .04$, CI[.00 .11]; $F(1,159)_{\text{single gender schools}} = 5.29$, $p < .023$; $\eta_p^2 = .023$, CI [0 .10]), such that greater testing group size and co-education schools were associated with higher judgements on the self-appraisal task overall (slope$_{\text{Testing group size}} = .02$, SE = .01, $p = .014$; contrast$_{\text{co-education – single gender}} = .26$, SE = .10, $p = .021$). Pubertal development ($F(1,159) = .46$, $p = .498$) and non-verbal reasoning ($F(1,158) = .51$, $p = .477$) were not related to judgements.

References

Ahmed, S. P., Piera Pi-Sunyer, B., Moses-Payne, M. E., Goddings, A. L., Speyer, L. G., Kuyken, W., Dalgleish, T., & Blakemore, S. J. (2024). The role of self-referential and social processing in the relationship between pubertal status and difficulties in mental health and emotion regulation in adolescent girls in the UK. *Developmental Science*. https://doi.org/10.1111/DESC.13503

Brysbaert, M., & Biemiller, A. (2017). Test-based age-of-acquisition norms for 44 thousand English word meanings. *Behavior Research Methods*, *49*(4), 1520–1523. https://doi.org/10.3758/S13428-016-0811-4

Kumle, L., Võ, M. L. H., & Draschkow, D. (2021). Estimating power in (generalized) linear mixed models: An open introduction and tutorial in R. *Behavior Research Methods*, *53*(6), 2528–2543. https://doi.org/10.3758/S13428-021-01546-0/FIGURES/8

Taylor, J. E., Beith, A., & Sereno, S. C. (2020). LexOPS: An R package and user interface for the controlled generation of word stimuli. *Behavior Research Methods*, *52*(6), 2372–2382. https://doi.org/10.3758/s13428-020-01389-1

Warriner, A. B., Kuperman, V., & Brysbaert, M. (2013). Norms of valence, arousal, and dominance for 13,915 English lemmas. *Behavior Research Methods*, *45*(4), 1191–1207. https://doi.org/10.3758/S13428-012-0314-X/FIGURES/12
